# Economic evaluation of a digital health intervention for preventing dementia in Canadians with mild cognitive impairment

**DOI:** 10.3389/fpubh.2025.1631676

**Published:** 2025-09-19

**Authors:** Seyed-Mohammad Fereshtehnejad, Karim Keshavjee, Peter C. Coyte

**Affiliations:** ^1^Division of Neurology, Faculty of Medicine, University of Toronto, Toronto, ON, Canada; ^2^Institute of Health Policy, Management and Evaluation (IHPME), Dalla Lana School of Public Health, University of Toronto, Toronto, ON, Canada; ^3^Division of Geriatrics, Department of Neurobiology, Care Sciences and Society (NVS), Karolinska Institute, Stockholm, Sweden

**Keywords:** dementia, prevention, digital health intervention, cost-effectiveness analyses (CEAs), mild cognitive impairment

## Abstract

Dementia poses an on-going Canadian challenge due to an aging population, with cases projected to rise significantly by 2050. This study evaluates the cost-effectiveness of a conceptual digital health intervention designed to prevent dementia in Canadians with mild cognitive impairment (MCI). The analysis is exploratory and conceptual, comparing different scenarios for the possible effectiveness of the digital intervention for dementia prevention. Using data from the Global Burden of Disease (GBD) 2021 study, a long-term economic evaluation was conducted from a healthcare payer perspective, comparing intervention costs to usual care between 2030 and 2050. Health outcome was assessed using disability-adjusted life years (DALYs) averted. The analysis revealed favorable incremental cost-effectiveness ratios (ICERs) well below conventional willingness-to-pay thresholds across all scenarios. Sensitivity analyses confirmed the robustness of these findings, underscoring the intervention’s potential to cost-effectively reduce dementia burden. The findings are based on modeled assumptions in the absence of empirical efficacy data and should therefore be interpreted with caution until validated in real-world settings. Yet, these results provide valuable insights for Canadian policymakers on scalable, proactive dementia prevention strategies.

## Introduction

Dementia is a progressive and irreversible condition, ranking among the leading worldwide causes of morbidity and mortality (1). The number of Canadians with dementia is projected to exceed 1.3 million by 2050, driven by population growth and aging (2, 3). Canada’s dementia care costs are projected to reach $153 billion by 2038, ranking as a top 10 cause of disability-adjusted life years (DALY) (3, 4). There is currently no definitive treatment for Alzheimer’s disease or most dementias (5). Protein-targeted agents have shown some promise as a possible disease-modifying therapy in recent clinical trials, but more data with longer follow-up are needed to fully understand their effectiveness (6).

The 2024 report from the *Lancet Standing Commission on Dementia Prevention* identifies 14 modifiable risk factors for Alzheimer’s disease and most dementias, emphasizing the need for proactive screening and intervention (7). Primary prevention, a proactive approach, relies on early intervention during the prodromal phase in individuals with mild cognitive impairment (MCI) 10–20 years before dementia diagnosis (8). MCI is a clinical diagnosis often seen as a transitional stage between normal aging and dementia (9). Recent clinical trials and quasi-experimental studies have shown that targeting these modifiable risk factors reduces the risk of developing dementia in the at-risk population (7, 10, 11). The Finnish Geriatric Intervention Study to Prevent Cognitive Impairment and Disability (FINGER) demonstrated that a 2-year multidomain intervention (diet, exercise, cognitive training, vascular risk monitoring) could improve or maintain cognitive functioning in individuals with MCI regardless of their socioeconomic or sociodemographic status (11, 12). Evidence from the Multi-domain Alzheimer Preventive Trial (MAPT) trial also supports the effectiveness of multidomain prevention strategies. In this 3-year randomized trial, older adults at increased dementia risk who received lifestyle interventions—with or without omega-3 supplementation—showed slower cognitive decline, particularly in orientation and delayed recall, compared with placebo (13). More recently, a digitally supported multimodal lifestyle program, the LETHE trial, has been designed to use the FINGER lifestyle program to promote brain health in Italy, Sweden, Finland and Austria (14). The feasibility study has revealed the potential of such digital intervention for older adults at risk of developing dementia to be applied in large-scale prevention programs (14).

This study introduces a conceptual framework for a digital health intervention (DHI) aimed at addressing modifiable risk factors for dementia in Canadians with mild cognitive impairment (MCI). Although the platform does not yet exist, it is envisioned to leverage machine learning and artificial intelligence (AI) to provide personalized recommendations for risk reduction and brain health promotion. By assessing the potential cost-effectiveness of such a digital intervention, this conceptual analysis compares anticipated costs with health outcome, measured in disability-adjusted life years (DALYs) averted, against the current reactive dementia care approach. The findings are designed to inform policymakers and stakeholders about the economic and public health implications of investing in innovative, proactive digital health solutions for dementia prevention in the future.

## Methods

### Model setting

- *Study Focus:* This economic evaluation investigates the cost-effectiveness of a conceptualized DHI compared to usual care—or status quo—in individuals with MCI to proactively prevent or postpone dementia in Canada.
- *Time Horizon:* The study uses a lifetime horizon considering the progressive and chronic nature of dementia and its long prodromal stage (8). To analyze the costs and health outcomes of dementia, cost effectiveness is calculated up to 2050.
- *Perspective:* The analysis is conducted from a healthcare payer perspective, emphasizing its relevance for policymakers tasked with optimizing resource allocation.
- *Target Population:* This evaluation focuses on Canadians aged 55 + with MCI, defined as cognitive decline beyond age expectations without major daily interference. Diagnostic criteria include subjective complaints, objective impairment, preserved independence, and absence of dementia, per NIA-AA Workgroups (15).

### Intervention

The conceptualized DHI is an innovative solution designed to mitigate the risk of dementia in individuals with MCI. This platform comprises two key modules:

a. *Mobile Application for Individuals with MCI*: The conceptual mobile app allows users to input and monitor data across multiple domains, including:

- Lifestyle: Habits and routines that may impact dementia risk.
- Cardiovascular Health: Metrics such as blood pressure, lipid profile, blood glucose.
- Diet: Tracking nutritional intake.
- Physical Activity: Exercise frequency and intensity.
- Mental Activity: Cognitive exercises and mental well-being practices.
- Sleep Parameters: Sleep patterns and quality.

These inputs once enriched with sociodemographic, comorbidities, genetics, and environmental data (if available) will enable the app to outline personalized risk profiles and strategies to promote brain health.

b. *Desktop Module for Care Providers:* This module allows healthcare professionals to review multidomain data, monitor patient risk profiles and progress in the advancement of brain health, and integrate information from electronic health records. Providers can use this interface to adjust intervention and management plans collaboratively with the individuals who may benefit from dementia prevention strategies.

At the core of the platform is a machine learning engine, augmented with generative AI. This central system analyzes integrated multidomain data to generate evidence-based personalized recommendations. The platform’s interventions target modifiable risk factors, aligning with guidelines for dementia prevention.

### Costs

- *Usual Care Costs for Dementia in Canada*: Dementia burden includes both direct (i.e., healthcare services, long-term care, out-of-pocket expenses) and indirect costs (i.e., productivity losses) due to unpaid caregiving. While healthcare payers mainly deal with only direct costs, to comprehensively assess the cost of dementia, both direct and indirect costs are quantified irrespective of who bares those costs. Historical data from the Canadian Center for Economic Analysis (16) provides a basis for estimating these costs over time (17).
- *Costs for the Digital Platform:* The costs of the DHI are informed by historical data from comparable digital health interventions (18) and expert opinion (19). These costs include development costs for integrating machine learning, generative AI, and compliance with healthcare data security standards, as well as operational costs for maintaining and scaling the platform. All costs are in 2024 Canadian dollars currency, $1.00 Can = $0.73US.

### Clinical outcome and data source

The main clinical outcome employed in this study is dementia-related DALY averted, which is a complementary health metric that evaluates the burden of disease. These health metrics are combined with the incremental costs of the intervention to generate incremental cost-effectiveness ratios (ICERs); namely, incremental costs per incremental DALYs averted. DALY, a global health metric, combines years of life lost due to premature mortality and years lived with disability (YLD) (20). Our analysis uses estimates of DALYs derived from the Global Burden of Disease (GBD) 2021 Study, which is further described in the next section. Updated estimates of DALYs from GBD 2021 were recently published in The *Lancet* in 2024 (21). Furthermore, the GBD Study offers forecasted DALY estimates for each country and disease, enabling insights into the future burden of diseases under current care standards and interventions up to the year 2050 (22, 23). For our study, DALY data specific to dementia in Canada was retrieved, covering both historical and projected trends (24).

### Global burden of disease study

The GBD study employs a comprehensive, systematic methodology to quantify the burden of diseases, injuries, and risk factors globally. It integrates data from diverse sources, including vital registration systems, population surveys, health facility records, disease registries, and published studies (25). To standardize and reconcile disparate data sources, the study uses advanced statistical methods such as DisMod-MR 2.1 designed specifically for GBD analyses (21). This method enables the estimation of consistent, comparable metrics across locations and time by synthesizing data, adjusting for biases, and filling gaps where direct observations are unavailable. The GBD framework includes rigorous cause-of-death modeling using tools like CODEm (Cause of Death Ensemble Model), which combines multiple predictive models to improve the accuracy and reliability of mortality estimates (24). The validity of GBD estimates is enhanced by combining rigorous methods with extensive data sources, standardized definitions, and advanced statistical tool that address potential biases and uncertainties. Regular updates and peer reviews further enhance the reliability and relevance of these estimates. The GBD study provides a wide array of health metrics, including life expectancy (24), healthy life expectancy (HALE), years of life lost (YLLs), years lived with disability (YLDs), and disability-adjusted life years (DALYs) (21). It also delivers comprehensive data on the prevalence and incidence of diseases and injuries, mortality rates, and the contribution of various risk factors to global health burdens (26). These metrics are stratified by age, sex, geography, and time, offering a detailed understanding of health trends worldwide. The GBD 2021 data are publicly available (27).

### Statistical analysis

- *Cost-Effectiveness:* The primary measure of cost-effectiveness in this study is the incremental cost-effectiveness ratio (ICER), calculated as the additional cost of DHI relative to usual care costs divided by the incremental number of DALYs averted with DHI relative to usual care. The ICER of the DHI was calculated under five different scenarios, assuming the DHI could reduce dementia incidence by 2, 5, 10, 20, and 50%. We modeled dementia incidence reductions from 2% (conservative) to 50% (optimistic) as scenario assumptions, not trial-based estimates. The upper range reflects projections from the Lancet Commission that up to 40–45% of dementia cases may be preventable through risk factor modification (7). The ICER was calculated as:
  $$\text{ICER}=\frac{C_{\mathrm{DHI}}-C_{\text{UsualCare}}}{\mathrm{DAL}Y_{\mathrm{DHI}}-\mathrm{DAL}Y_{\text{UsualCare}}}$$
- *Willingness-to-Pay Thresholds:* The study evaluates the cost-effectiveness of the digital health intervention using conventional willingness-to-pay (WTP) thresholds, which reflect the maximum amount a healthcare payer is willing to pay for one DALY averted. Based on GBD estimates, the WTP threshold for countries with a very high human development index (HDI), like Canada, is $69,499 USD or $97,274 CAD (28). An ICER below these thresholds indicates the intervention is economically justifiable.
- *One-Way Sensitivity Analysis:* A one-way sensitivity analysis assessed the robustness of cost-effectiveness results by varying parameters like intervention costs, effectiveness, and DALY estimates within 20%. Results highlight parameters with the greatest impact on ICER and uncertainties.
- *Discount rate:* In our analysis, we applied a 1.5% annual discount rate to both costs and health outcomes, as recommended by the Canada’s Drug Agency (formerly the Canadian Agency for Drugs and Technologies in Health, CADTH) (29). This approach accounts for the time preference of costs and benefits, ensuring a consistent valuation of future outcomes and aligning the analysis with standard practices for economic evaluations in Canada.
- *Maximum Cost of the Intervention Program:* Another sensitivity analysis was performed to determine the maximum allowable cost of the digital health intervention that would still result in an ICER below the WTP threshold. By iteratively increasing the intervention cost and recalculating the ICER, this analysis provides insights into the budgetary limits for scaling the intervention while maintaining cost-effectiveness.

## Results

### Trajectories of dementia prevalence, burden and costs in Canada

**Figure 1:**
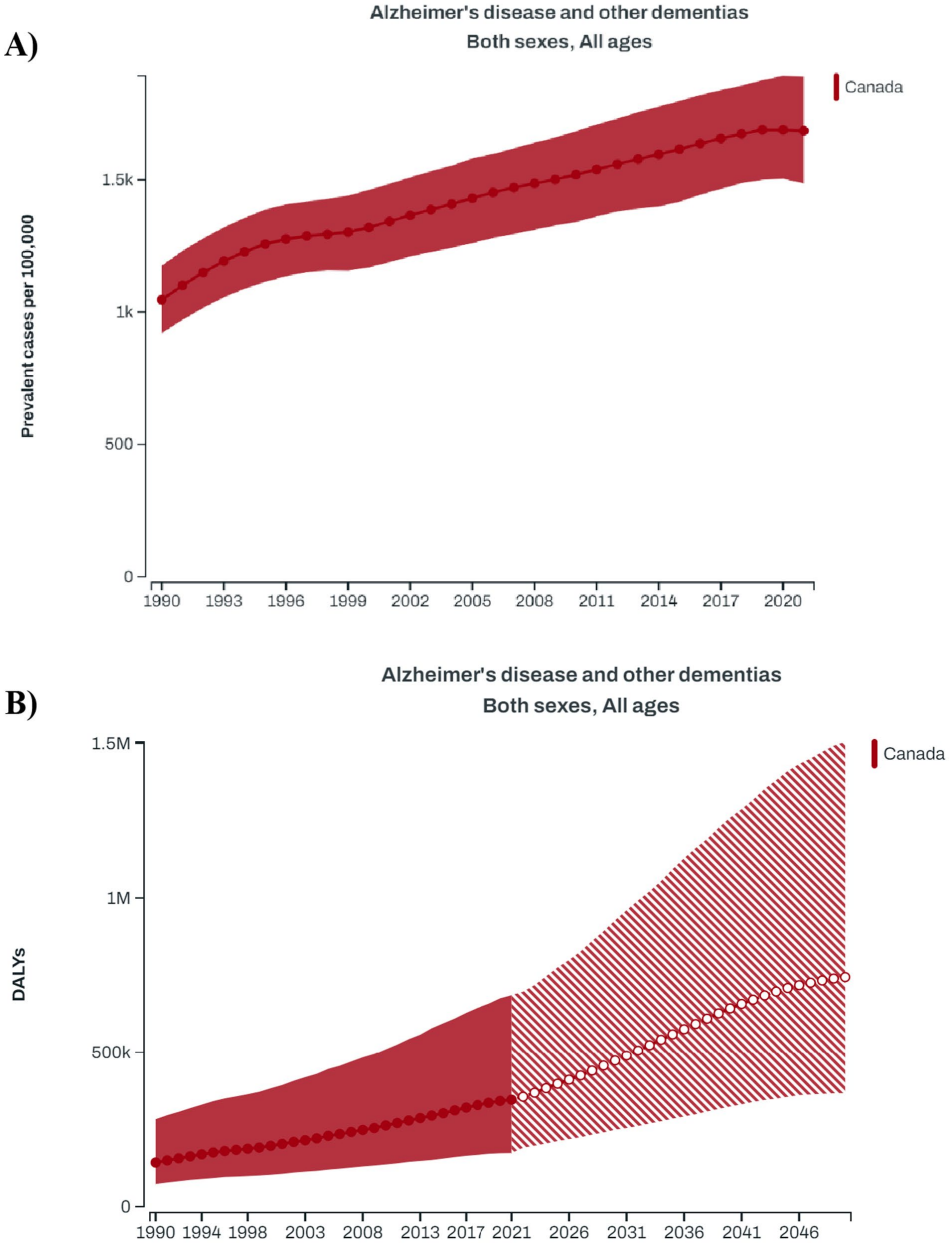
**(A)** Longitudinal trends in the prevalence of dementia in Canadian population during 1990–2021; **(B)** forecasted trend for the disability-adjusted life year (DALY) attributed to dementia in Canada during 1990–2050 (Data source: Global Burden of Disease Study).

As per the GBD 2021 estimates, there has been a steady increase in the prevalence of dementia in Canada over the last three decades 1990–2021 (3) (Figure 1A). According to the GBD foresight (23), the total DALYs associated with dementia in Canada will rise from the current estimate of 985.1 per 100,000 population (95% UI: 512.8–1975.8) in 2024 to 1524.4 (95% UI: 744.3–3119.6) per 100,000 population in 2050 (Figure 1B). The estimated costs of dementia care in Canada, based on projections for 2020 to 2050, would increase from $40.1 billion in 2020 to $110.3 billion by 2050, encompassing both direct (40.2%) and indirect (59.8%) costs (Table 1).

**Table 1:** Projected economic and disability burden of dementia in Canada (2020–2050), based on forecasted incidence and prevalence trends.

Cost component		2020	2030	2040	2050
Direct Costs	Emergency Department	$40	$70	$90	$100
	Hospitalizations	$6,440	$10,620	$15,270	$18,020
	Home care	$310	$510	$610	$630
	Long-term care	$6,990	$11,680	$17,760	$21,800
	Out of pocket	$1,360	$2,250	$3,230	$3,810
Productivity	Caregivers	$21,830	$35,990	$52,360	$60,730
	Dementia patients	$3,160	$4,130	$4,510	$5,190
Total ($M)		$40,130	$65,230	$93,830	$110,280
Dementia Prevalence (/100,000)		1688.45	-	-	3695.65
DALYs (/100,000)		920.09	1136.95	1408.12	1524.39

M, Million; DALY, Disability-adjusted life year; Data sources: Global Burden of Diseases (GBD) Study, Alzheimer’s Society of Canada, Canadian Center for Economic Analysis.

### Intervention costs and incremental costs

The digital intervention’s costs are projected to increase from $0.45 million initially to $3.35 million by 2050 due to scaling and maintenance (Table 2). Cost-effectiveness analysis considered three scenarios associated with the introduction of the digital health intervention with 2, 5, and 10% reductions in dementia incidence compared to usual care. These conservative rates of reduction reflect variability in the literature but are notably lower than the *Lancet Commission*’s estimate that multidomain interventions could prevent up to 40% of dementia cases (30). The results are summarized in Table 3.

**Table 2:** Estimated costs for developing and maintaining digital health intervention for dementia prevention.

Category	Details	Estimated cost
Development Costs	Basic app features (*UI, data input forms, backend server*)	$30,000–$50,000
	Advanced features (*ML integration, real-time analytics*)	$50,000–$100,000
	HIPAA/PHIPA compliance (*data security and privacy*)	$10,000–$25,000
Data Management and Integration	Connecting to external data sources (e.g.*, EHRs, health APIs*)	$20,000–$40,000
	Cloud hosting and data storage (e.g.*, AWS, Google Cloud*)	$5,000–$10,000/year
Machine Learning Development	Developing and training ML models	$20,000–$50,000
	Continuous updates and retraining for new data	$10,000–$30,000/year
Testing and Validation	User acceptance, performance, and regulatory validation	$10,000–$20,000
Maintenance and Updates	App updates, bug fixes, and new features	$10,000–$25,000/year
Total Development Cost (One-Time)		$165,000–$350,000
Annual Maintenance Costs		$70,000–$100,000/year

UI, User Interface; ML, Machine Learning; HIPAA, Health Insurance Portability and Accountability Act; PHIPA, Personal Health Information Protection Act; EHR, Electronic Health Record; AWS, Amazon Web Services.

**Table 3:** Cost-effectiveness scenarios for a digital health intervention to prevent dementia in Canada (bolded values indicate cost per DALY averted below the $97,274 threshold).

Year	Reduction in dementia incidence *(%)*	Usual care costs (Discounted) *(Million $)*	Intervention costs (Discounted) *(Million $)*	Usual care DALYs *(per 100 k)*	Averted DALYs *(per 100 k)*	Incremental cost per discounted DALY Averted *($)*
2030	2%	65,230 (base year)	1.35 (base year)	1136.95	22.74	59,300
	5%	65,230 (base year)	1.35 (base year)	1136.95	56.85	23,700
	10%	65,230 (base year)	1.35 (base year)	1136.95	113.70	11,900
	20%	65,230 (base year)	1.35 (base year)	1136.95	227.39	5,940
	40%	65,230 (base year)	1.35 (base year)	1136.95	454.78	2,970
2040	2%	93,830 (80800)	2.35 (2.02)	1408.12	28.16	83,300
	5%	93,830 (80800)	2.35 (2.02)	1408.12	70.41	33,400
	10%	93,830 (80800)	2.35 (2.02)	1408.12	140.81	16,700
	20%	93,830 (80800)	2.35 (2.02)	1408.12	281.62	8,340
	40%	93,830 (80800)	2.35 (2.02)	1408.12	563.25	4,170
2050	2%	110,280 (81763)	3.35 (2.48)	1524.39	30.49	110,000
	5%	110,280 (81763)	3.35 (2.48)	1524.39	76.22	43,900
	10%	110,280 (81763)	3.35 (2.48)	1524.39	152.44	22,000
	20%	110,280 (81763)	3.35 (2.48)	1524.39	304.88	10,980
	40%	110,280 (81763)	3.35 (2.48)	1524.39	609.76	5,500

DALY, Disability-adjusted life year.

### DALYs averted and cost per averted DALY (ICER)

The DALYs attributed to dementia under usual care are projected to increase from 920.09 per 100,000 population in 2020 to 1,524.39 per 100,000 population in 2050. The digital health intervention scenarios are expected to yield varying reductions in DALYs (Table 3). While a 2% reduction prevents 22.7 to 30.5 DALYs per 100,000 population over the two decades from 2030 to 2050, a 10% reduction may prevent between 113.7 to 152.4 DALYs per 100,000 population. The ICER was calculated as the incremental cost per DALY averted. Accordingly, the ICERs vary by year and intervention effectiveness, as speculated by the percentage of reduction in dementia prevalence (Table 3). For a 2% reduction, the incremental cost per DALY averted ranges from $59,300 in 2030 to $110,000 in 2050, and with a 10% reduction the ICERs are considerably smaller and range from $11,900 per DALY averted in 2030 to $22,000 per DALY averted in 2050.

The findings show that the intervention becomes more cost-effective with greater dementia incidence reductions. Despite rising costs and DALY reductions over time, the ICER stays below the $97,274 WTP threshold in all but the least effective scenario and then only in 2050 (2% reduction by 2050).

### Sensitivity analysis

The one-way sensitivity analysis evaluates the impact of variations in key parameters on the incremental cost per averted DALY. Variation in three parameters were analyzed: usual care costs, intervention costs, and DALYs averted, each varied by 20% from their base values (Table 4). Among these, the number of DALYs averted had the greatest impact, resulting in a ± 0.005 million $ change in the cost per averted DALY relative to the base case ICER. Variations in intervention costs had a slightly smaller impact, causing a ± 0.004 million $ shift in the cost per averted DALY when compared to the base case ICER.

**Table 4:** One-way sensitivity analysis for the cost-effectiveness of digital health intervention for dementia prevention in Canada.

Parameter	Base value	+20% impact	-20% impact	Change in cost per averted DALY *(Million $)*
Usual Care Costs *(Million $)*	65,230	78,276	52,184	±0.002
Intervention Costs *(Million $)*	1.35	1.62	1.08	±0.004
DALYs Averted *(per 100 k)*	56.85	68.22	45.48	±0.005

**Figure 2:**
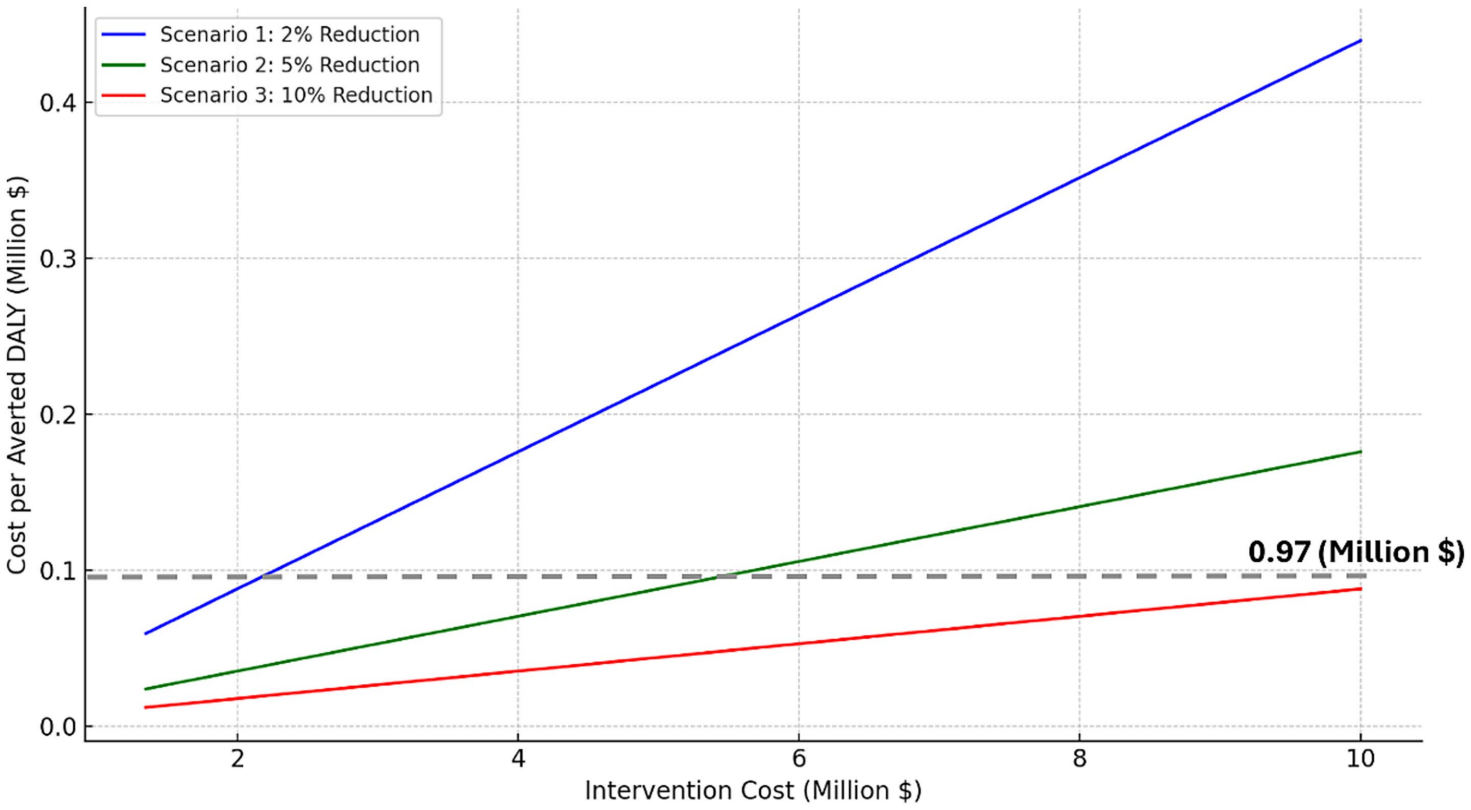
Modeled cost per DALY averted under dementia prevention scenarios (2, 5, 10% reduction), with dotted line indicating Canada’s willingness-to-pay threshold.

The second sensitivity analysis explores how varying the cost of the digital health intervention affects the incremental cost per averted DALY under three dementia incidence reduction scenarios (2, 5, and 10%; Figure 2). As intervention costs increase, the incremental cost per averted DALY rises, showing diminishing returns on health outcomes. In the 10% reduction scenario, the incremental cost per averted DALY remains below the WTP threshold even when the intervention costs were increased to $10 million. In contrast, the 2% reduction scenario only stays below WTP threshold if intervention costs remain below $2.2 million. All analyses and modeling were conducted using Microsoft Excel (Microsoft Corp., Redmond, WA) for base-case calculations and scenario modeling, and Python 3.11 (Python Software Foundation, Wilmington, DE) for sensitivity analyses and visualizations using standard data science libraries (e.g., pandas, NumPy, matplotlib).

## Discussion

This study demonstrates the potential of a digital intervention to reduce the burden of dementia in Canada in a cost-effective manner. The intervention shows increasing cost-effectiveness with higher hypothesized reductions in dementia incidence. Even under conservative assumptions of modest reductions (i.e., 2–10%)—considerably below the 40% potential estimated by the *Lancet Commission* (7, 30)—, the digital health intervention could potentially deliver significant health benefits, reducing the burden of dementia as measured by DALYs. Importantly, the intervention remains cost-effective across most scenarios when compared to conventional WTP thresholds for averted DALYs (28). All costs and health outcomes were discounted at an annual rate of 1.5%, consistent with CADTH guidelines for economic evaluations. This ensures comparability across studies and reflects the time preference for costs and benefits in the Canadian context. Sensitivity analyses show that intervention costs and health outcomes influence the cost-effectiveness of the digital health intervention, but overall conclusions remain consistent. We did not perform a probabilistic sensitivity analysis because of the lack of empirical data to define valid probability distributions for many key parameters in this emerging area.

Emerging evidence shows that interventions targeting modifiable dementia risk factors can be cost-effective (31). Research indicates that reducing dementia risk by 5% through well-designed, sustained programs is cost-effective, even with relatively high per-person costs of such interventions (32). A scoping review of seven studies analyzing multidomain dementia prevention interventions found that targeting modifiable risk factors in at-risk populations is generally cost-effective, with interventions yielding an average gain in quality-adjusted life year (QALYs) of 0.08, costs averaging 472.20 Euros per person, and ICERs ranging from cost-saving to 104,189.82 Euros per QALY (33). Like our proposed intervention, the LETHE project has recently been implemented in the EU, as a multidomain digital platform targeting dementia risk in at-risk individuals (14). The LETHE study adapts the Finnish FINGER multidomain lifestyle intervention (11) into a digitally supported format, delivered across four European countries (Austria, Finland, Italy, Sweden) (14). It integrates in-person professional activities with an Android mobile application (the LETHE App), supported by wearables for passive data collection. The LETHE program integrates a structured lifestyle approach targeting modifiable risk factors like diet, exercise, cognitive training, and sleep management. Primary outcomes include feasibility measures such as retention and adherence, as well as change in validated dementia risk scores. Early results are promising; 156 at-risk older adults were randomized, and retention at 6 months was 98.7%, with daily app use reported by about 50% of intervention participants. These preliminary findings demonstrate that digitally supported multimodal dementia prevention programs are feasible, well accepted, and usable in older adults—a key precedent for our proposed Canadian model (14). Innovations like the ‘Digitized Memory Clinic’ highlight the transformative role of digital technologies in managing dementia risk, offering individualized prognostication and personalized prevention (34). Our analysis shows that such digital interventions for dementia prevention in Canada may be cost-effective. Remaining reactive in dementia care incurs significantly higher costs, as highlighted by recent findings that even advanced anti-amyloid treatments, such as Lecanemab (35), are not cost-effective compared to usual care for patients with MCI or mild dementia unless priced substantially below current levels (36). Noteworthy, the use of digital tools and AI in dementia prevention raises ethical considerations. Potential risks include false reassurance if predictions are overly optimistic, algorithmic bias due to underrepresentation of certain groups in training data, and challenges related to transparency and accountability (37). These concerns highlight the importance of rigorous validation, equitable design, and regulatory oversight in the development and deployment of AI-driven prevention tools.

Another important factor to consider is the feasibility of implementing such a digital intervention, especially in the Canadian context. Digital access and health literacy are not consistent throughout Canada due to its large geographic area, notable rural–urban disparities, and diverse ethnic and cultural groups, including Indigenous communities and sizable immigrant populations (38). Disparities in language, socioeconomic background, and cultural perspectives on technology may have an impact on adoption and continued use (39). To guarantee that such interventions are available and successful across the country, it is crucial to customize implementation strategies, co-design with diverse communities, and follow equity-focused policies.

Our study has several limitations. The economic evaluation relied on historical data and modeled assumptions, which may limit the robustness and generalizability of the findings. The absence of empirical data introduces inherent uncertainty regarding the intervention’s effectiveness and feasibility. We only calculated cost-effectiveness in our study, as the lack of data on dementia-specific utility estimates in Canadian population made cost-utility modeling based on QALYs impractical. Another key limitation of this analysis is the absence of a validation or feasibility study of the proposed digital health intervention in the Canadian context. While our economic evaluation provides modeled estimates of potential cost-effectiveness, the platform itself remains conceptual and untested in real-world settings. Platform maintenance costs were incorporated into our model, however, the potential impact of evolving data regulations and long-term variability in healthcare costs was not explicitly modeled and remains an area for future analysis. Our model did not incorporate user adherence, attrition, or provider engagement, which are well-recognized factors influencing the real-world impact of digital interventions. Nevertheless, early data from the LETHE trial are promising, with nearly full retention and high adherence at 6 months (14), indicating that older adults can successfully engage with digitally supported multidomain prevention programs. Additionally, disparities in healthcare access, digital health literacy, and variations by ethnicity, place of residence (rural versus urban), and socioeconomic status were not explicitly modeled due to the absence of subgroup-specific data, which may have influenced the projected outcomes. The analysis does not account for future changes in healthcare costs, AI upkeep, data privacy laws or the emergence of novel dementia treatments by 2050, further contributing to the uncertainty. Another limitation is the absence of a comprehensive probabilistic sensitivity analysis, which would have allowed for a more rigorous assessment of the robustness of the base case findings. Nonetheless, under reasonable distributional assumptions, it is unlikely that such an analysis would have fundamentally altered the primary conclusions. Future studies with empirical data collection are essential to validate these findings and strengthen the evidence base for digital health solutions.

This cost-effectiveness analysis shows that a proactive, digitally supported health intervention to reduce dementia risk can be cost-effective, especially with greater reductions in dementia incidence. All estimates in this study are modeled at the national level using per-100,000 population rates, based on dementia prevalence and burden forecasts from the GBD 2021 Study, applied to the Canadian population aged 55 and older. The findings suggest such interventions could significantly reduce dementia’s burden in Canada, even with rising intervention costs and conservative efficacy estimates. While the current analysis offers a high-level evaluation, future studies should incorporate detailed modeling of disease progression and intervention effects on various stages of dementia to refine cost-effectiveness estimates. Despite these limitations, the results support the implementation of scalable, evidence-based digital health platforms as a promising approach to address the rising dementia burden in Canada, urging policymakers to invest in proactive strategies for dementia prevention.

## Data Availability

The original contributions presented in the study are included in the article/supplementary material, further inquiries can be directed to the corresponding author.
